# Letter from Dr. Baker

**Published:** 1846-06

**Authors:** E. Baker

**Affiliations:** No. 6 Warren-st., N. Y.


### ARTICLE V.

### *Letter from* Dr. Baker.

It is certainly not a very pleasant duty when a portion of the members of any society, find it necessary to vindicate and justify themselves in a course of conduct, at variance with the opinion of a majority of the same. I shall write in the plural number, because I am morally certain, that in doing so, I am expressing the sentiments, as far as I am able, of those whose opinions on this subject, accord generally with my own.

It is very well known by every member of the American Society of Surgeon Dentists, that the subject of amalgam, has occupied its attention, more or less, for several consecutive years at its general meetings. A majority of it, present at the time, have resolved repeatedly, that it is a worthless and dangerous article when used in filling teeth, and have declared it *malpractice* to use it under any form, or in any case whatever. No one will dispute this position, as to its *general* use, but as in most general rules, there are some exceptions, so in this. There is, perhaps, a majority of the members of the Society, living in the city of New York, who are as much opposed to its *indiscriminate* and unskilful use as any portion of the Society, yet they believe, under certain circumstances and in certain cases, it is not only useful, but the "very best" filling that is now in use. It is perhaps unnecessary to go into a description of those *circumstances* and *cases* at this time.

At length the Society, at its last general meeting, passed a resolution directing the secretary to issue certificates or pledges in accordance with said resolutions, to each and every member of the Society, requiring him to sign the same and return it to the secretary, and for non-compliance thereto his name was ordered to be stricken from the roll of members.

The *substance* of the pledge, issued for the members to sign and return the same to the secretary, was to this effect, viz. that amalgam, in any of its preparations whatever, was a worthless and dangerous material for filling teeth, in *any case* whatever, and declaring it to be *malpractice* to use the same in *any* case.

Now it is very plain, that those of us who had, with the greatest success, used this composition, "in certain cases," and who believe, also, that they have the natural, inherent and absolute right to follow the promptings of *their own* judgments as it respects practice, could not sign such a paper, for reasons which are self-evident. From a non-compliance with the terms of the aforesaid resolutions, a number of us, who refused to comply, are under the *ban*, or in the intermediate state, (or purgatory,) or completely cut off or expelled.

If by "striking off the roll" means expulsion, we shall enter our solemn protest against such an act, believing *that* question to be still open, and that no member can be expelled except in a constitutional manner, by a constitutional majority. It will be time enough when the day of trial comes, (if it ever come,) to present arguments.

Suffice it to say, that a constitution is a system of fundamental rules, principles and ordinances, for the government of a nation, state or *society.* And where there is any pretension to freedom, the constitution is *paramount* to the statutes or laws enacted by a legislature, limiting and controlling the power of the legislative body. The constitution is a *particular* law, ordinance or regulation, made by the authority of a *superior* power. It therefore appears that a body of men, forming a constitution, act in a different capacity entirely from one assembled for the purpose of legislation. Our constitution or supreme power prescribes under what circumstances, and by what majority, a member of our Society shall be *expelled.*

Hence the legislative or inferior power has the authority only to produce charges, specifications, arraign and try members in a constitutional manner. If those who formed and adopted the constitution of our Society, had meant that the power of expelling should rest entirely, or at all, with the members in their legislative capacity, no constitutional provision would have been made for that purpose. I am induced to make these remarks because some have supposed that to be "stricken off the roll" means expulsion. But it seems most reasonable that the true construction cannot amount to any thing more than to be under the *ban* of the Society, or in an *intermediate* state.

If it is said that we stand expelled according to *law,* we reply that no law is binding when it encroaches on our natural, unalienable or constitutional rights; and a *resolution* is nothing more than an opinion or fixed purpose of mind, and is of no *legal* force whatever, until it is succeeded by "Be it enacted, &c." Now the only safety to freedom is strict construction of constitution and laws.

The stretch of power assumed by the Society at its last meeting seems to have attracted the attention of the State Society of Dentists of Virginia, when they say, "We claim no authority over the opinions of our members," or "require any pledges other than those which exist among honorable men," or "conceding that the society was not a court of conscience," &c. and they might have truly said, also, that to *assert* or *vote* a thing to be *malpractice* in *all* cases whatever, is very different from *proving* it to be so, which the constitution will require before any member can be legally expelled on a charge of that kind.

Members of our Society, living in Virginia, whose state arms are represented by Liberty treading on a tyrant and his chains, and whose motto is "Sic semper tyrannis," will arouse at the least encroachment on liberty or right.

It will be recollected, that, at the last meeting of the Society, for the sake of restoring harmony and maintaining peace in the Society, and it being represented that many ignorant practitioners of dentistry were making an indiscriminate use of amalgam, most of us in this city agreed to suspend the use of it altogether, for the present. It cannot be denied that this was not a great concession on our part, and, as we think, should have satisfied the Society. We even offered to sign a pledge to use it no more. Here we went farther than our obligations to truth and duty required. But when it was required that we should sign a pledge such as was afterwards sent to us, to that we could not submit; had we done so, our degradation would have been complete.

So the horns of the dilemma were—sign the pledge or have your names stricken from the roll of members. The result has shown which horn we chose, and we abide the result. Nobody disputes but what a majority can exclude a minority under any circumstances, for a minority, who respect themselves, will certainly not contend against "brute force," for so any act may be regarded, that is not founded on law or usage.

The opinion of a member who refused to sign the pledge, that "the Society has transcended its powers, and violated the compact which ushered it into existence, by enacting resolutions which are arbitrary, unjust and unconstitutional," and that of another, who says, "the Society has certainly transcended its powers," &c. evidently alluded to the *constitution*, when expressing those opinions, and in this sense they mean to be understood, when they say, the Society has no *right* to pass such resolutions or *require* such pledges, because it cannot constitutionally enforce such acts. Now it is very evident that the junior editor, Dr. Westcott, means the "*brute force*" *right*, when he says, "In respect to this question of *right*, we have yet to learn that a voluntary association, untrammelled even by a *charter*, as is the American Society of Dental Surgeons, has not a right to pass *any* measure they please, even to the extent of expulsion of their own members." "But if our objectors mean, by saying that the American Society had no right to pass the offensive resolutions referred to, that those resolutions are unreasonable and unjust, this constitutes entirely a *new* issue."

*We* not only think those resolutions and pledges *unreasonable* and *unjust*, but we regard them as a "brutum fulmen," mere paper bullets, and which cannot be constitutionally enforced, and on these grounds we *place* and are ready to *stand* the *issue.*

Does the junior editor mean to be understood, that because the Society is *not* chartered, it is at liberty to commit acts which in a chartered society would be considered unlawful, oppressive and ridiculous? We really believe the Society will not sanction such reasoning.

Although I am well satisfied in my *own* mind, by actual experiment and trial, of the utility of amalgam in certain cases, yet I may find it difficult to satisfy the minds of some members in our Society, to the same extent, if at all. And as it is said, "a prophet is not regarded in his own country," I will introduce an *authority* living in Paris, who may be said to be at the head of his profession, and who undertakes to speak of, and who is well acquainted with, the practice of the principal dentists in Europe.

PARIS, RUE DE LA PAIX, No. 11,
*23d February,* 1846.

MY DEAR BAKER,

Your favor of the 9th Jan., asking my opinion on the use of an amalgam of silver and quicksilver for filling teeth, "in certain cases," is at hand.

I reply, I *was* among those who thundered forth universal condemnation against the use of this article. I *am* among those who believe that, "in certain cases," it is very useful, *and I act accordingly.* I have lived too long in the world, seen and had too much practice in our profession, to now *universally* condemn an article, that is used in some cases, (I believe,) by every respectable dentist in Europe, at least I do not know of a single exception.

That there have been, and that there yet exist, many unprincipled charlatans, who, by the indiscriminate use of this composition, often give their patients much pain without any benefit, I do not deny. There are also many respectable dentists who use this composition in cases where you or myself would use gold. Yet to condemn the use of amalgam in *all* cases, merely because its use is abused in *some*, I think unwise.

The unprincipled quacks who first introduced its use with you, whose only object was to pocket the money of a credulous class of patients, did, undoubtedly, do much injury; but we see cases of suffering and injury, from the use of gold, of the file, and even of extracting teeth, yet who will dare to say that each and all of these are not useful and necessary "in certain cases?"

I am fully aware, that in the opinion that I give you relative to the use of amalgam for filling teeth, that many dentists in America, whose opinions I respect, whose talents I admire, and whose friendship I cherish, think very differently from me; but as you ask my opinions, I give them, and my *practice* is in accordance with my principles.

My observation is, that much good has been and may be done by a judicious use of this composition. Much injury has been and will continue to be done, by an indiscriminate, unprincipled use of this amalgam.

I am sorry to see so many of our first dentists in America condemn, in *all* cases, the use of this composition; for I believe, were they, with their ability and judgment, to use it as it is used by *some* dentists in Europe, then they would agree with me, that the article "has been more sinned against than that it has sinned."

Neither you nor I can prevent chalatanism nor imposition from gaining proselytes; we have but to do that which our experience and our ideas of rectitude demand, &c.

I hope ere long to pay you a visit, when we will talk over those things; in the interim I shall be most happy to hear from you.

I am, very truly, yours, &c,

C. L. BREWSTER.

I should be happy could I now close my observations on this subject; but, for a variety of reasons, I am compelled to notice what appears to me to be objectionable, both in manner, matter, and a statement of facts. I have no doubt the writer stated what he had heard from others, but he had been misinformed in many respects.

If I had pursued the devious course he represents, I should feel myself fully justified in my present opinion, for I hold when the evidences concerning common things change, a man should change his course of conduct.

The circumstances to which I allude are these:—The junior editor, Dr. Westcott, in Miscellaneous Notices, which appeared in the December number of the Journal, while expatiating on the troubles, anxieties, and the action the Society had had for several years on amalgam, after giving the Virginia Society quite a lecture, for a very small appearance of what he would call contumacy, and in order to enforce, (as I suppose,) the necessity of resorting to those coercive measures adopted at the last general meeting, he thought fit to make use of me in a manner which I do not approve of. In the first place, I do not think the Journal should be the medium for arraigning any person for his conduct, but should be applied to the purpose to which it was intended. In the next place, he makes a *covert* attack on me, which certainly is not very respectful. This consists in publishing my name in small capitals, and mentioning various circumstances, so that I may be identified. "We find *him* (the Dr. means E. Baker) one of the foremost in carrying out measures to expel, not from society, but from the country, Monsieur Mallan, for the malpractice of using mineral paste." Here the doctor is entirely mistaken; I took no part for nor against the Mallans. It is possible this mistake may have arisen from the circumstance of my having joined a number of my brethren, some years previous, in a similar crusade against the Crawcours, who, like the Mallans, were impostors. Since then, I have not "run a tilt or a muck" against quacks or amalgam; I neither think it policy or respectable to do so. I was one of the committee in Philadelphia, but took no active part, the chairman reported what he pleased. To be sure I was afterwards in New

York, and filled a few teeth with amalgam, and found it most excellent "in certain cases." It is very likely I gave Dr. Bliss a certificate to the same effect, and if I were to give another, it would be the same. I have pursued no inconsistent course, as would be inferred from Dr. Westcott's account. To be sure, I never "thundered" against amalgam like my friend Brewster, but my first impressions were against it, which have changed gradually, till I am convinced amalgam is useful "in certain cases," and there my opinion will probably rest. It is an old but trite saying, that "wise men *change* their minds, but fools *never* do." How many things which have been received as truths, have afterwards been found to be false, and how many things, which at the time have been ridiculed and disbelieved, have afterwards proved to be true.

There perhaps has been more intolerance, illiberality, disputation and ill blood exhibited in the conduct of members of the healing profession, than any other. This arises partly from the nature of the subject, and partly from ignorance and prejudice. Witness some of the greatest discoveries in medicine, and other discoveries which have almost banished disease; and strange to tell, those discoverers and benefactors of mankind have, in their turn, been ridiculed, traduced, and even persecuted. Nothing could exceed the virulence of a great portion of the practicing physicians, at the time, against the introduction of *inoculation* for the small pox. Dr. Boylston's house, of Boston, was mobbed, occasioned by introducing inoculation. With what opposition did Jenner meet; and, if necessary, many other instances could be mentioned. I will only advert to the case of the great Sydenham.

"His towering genius being too elevated for appreciation by the College of Physicians and his shallow colleagues, they endeavored to banish him, as guilty of medical heresy, *out* of that illustrious society, (the College.*) Sydenham, though a graduate of Cambridge, and a fellow of Oxford, was not deemed worthy of the fellowship, but was cast down to the inferior position of licentiate, by a host of moral pigmies. Licentiates in

* Farr's History.

those days consisted of oculists and aurists, and other individuals, who devoted themselves to particular branches, and who in these times appear to have possessed an inferior degree of education.

The remark of Sydenham, in reference to his persecutors, was worthy of his genius. "It is better to *assist* mankind, than to be COMMENDED by them." Many other *pictorials* or *illustrations* could be brought forward, but perhaps this will be sufficient.

Now, how is it, that *we* do not see as much virulence and prejudice among physicians, and persecution by corporate bodies of physicians of the present day, as there was formerly? Are not these the principal reasons? They have become *less* prejudiced, *more* enlightened, and liberality and wisdom follow, as a matter of course.

E. BAKER,

*May 4th*, 1846. No. 6 Warren-st., N. Y.

---

